# A New α_5_β_1_ Integrin-Dependent Survival Pathway Through GSK3β Activation in Leukemic Cells

**DOI:** 10.1371/journal.pone.0009807

**Published:** 2010-03-23

**Authors:** Fabienne De Toni-Costes, Mathieu Despeaux, Jessica Bertrand, Ezzeddine Bourogaa, Loïc Ysebaert, Bernard Payrastre, Claire Racaud-Sultan

**Affiliations:** 1 Unité 563, Institut National des Sciences et de la Recherche Médicale, Toulouse, France; 2 Université Toulouse III Paul-Sabatier, Centre de Physiopathologie de Toulouse Purpan, Toulouse, France; 3 Centre Hospitalier Universitaire de Toulouse, Hôpital Purpan, Service d'Hématologie, Toulouse, France; INSERM U567, Institut Cochin, France

## Abstract

**Background:**

Cell survival mediated by integrin engagement has been implicated in cell adhesion-mediated drug resistance. We have recently demonstrated that the activation of glycogen synthase kinase 3 β (GSK3β) is a new pathway supporting the chemoresistance of leukemic cells adhered to fibronectin.

**Methodology and Principal Findings:**

We show here that in conditions of serum starvation, the fibronectin receptor α_5_β_1_ integrin, but not α_4_β_1_, induced activation of GSK3β through Ser-9 dephosphorylation in adherent U937 cells. The GSK3β-dependent survival pathway occurred in adherent leukemic cells from patients but not in the HL-60 and KG1 cell lines. In adhesion, activated GSK3β was found in the cytosol/plasma membrane compartment and was co-immunoprecipitated with α_5_ integrin, the phosphatase PP2A and the scaffolding protein RACK1. PP2A and its regulatory subunit B' regulated the Ser-9 phosphorylation of GSK3β. In adherent leukemic cells, α_5_β_1_ integrin but not α_4_β_1_ upregulated the resistance to TNFα-induced apoptosis. Both extrinsic and intrinsic apoptotic pathways were under the control of α_5_β_1_ and GSK3β.

**Conclusions and Significance:**

Our data show that, upon serum starvation, α_5_β_1_ integrin engagement could regulate specific pro-survival functions through the activation of GSK3β.

## Introduction

The glycogen synthase kinase 3β (GSK3β) is a serine/threonine protein kinase that is involved in many physiological processes, playing important roles in glucose metabolism, cell cycle division, cell adhesion and apoptosis. Deregulation of GSK3β activity is implicated in the pathogenesis of neurodegenerative and metabolic disorders, but also in cancer [1]. GSK3β is constitutively active under its Tyr-216 phosphorylated form and regulates many intracellular signaling pathways. At the post-translational level, the function of GSK3β is inhibited through phosphorylation of the Ser 9 residue by other protein kinases, including Akt, in response to insulin and growth factors [2].

Following integrin engagement, both inhibition and activation of GSK3β have been described. GSK-3β is inhibited by Ser-9 phosphorylation by the ILK/Akt and Cdc42/PKCζ pathways to promote integrin-mediated cell proliferation or migration, respectively [3], [4]. Conversely, cell adhesion to a 3D collagen matrix through α_2_β_1_ engagement promotes activation of GSK3β as well as protein phosphatase 2A (PP2A) [5]. PP2A has been previously shown to reactivate GSK3β through dephosphorylation of Ser-9 [6], [7]. However, no role has been ascribed to the activated form of GSK3β downstream of integrin engagement.

We have previously shown that GSK3β activation promotes the chemoresistance of adherent leukemic cells on fibronectin or on osteoblasts under serum starvation [8]. The endosteal niche supports chemoresistant leukemic stem cells [9] and is thought to be rich in fibronectin and hypoxic [10]. Adhesion of serum-starved leukemic cells to fibronectin through α_4_β_1_ and α_5_β_1_ engagement allows both Ser-9 dephosphorylation of GSK3β and NF-κB activation [8]. Others and we have demonstrated that GSK3β can upregulate cell survival through epigenetic and IkB-independent control of NF-κB activity [8], [11]–[14]. Strikingly, the anti-apoptotic role of GSK3β has been demonstrated in different tumors and may involve resistance to death receptor-induced apoptosis [15]–[20]. Recently, GSK3β was found associated with DDX3 and c-IAP-1 in a death antagonizing signaling complex at death receptors and the resistance to apoptosis was overcome by GSK3 inhibitors [21]. A mitochondrial-mediated cell death was also found regulated by GSK3 [22].

Adhesion to fibronectin through α_4_β_1_ and α_5_β_1_ engagement supports cell adhesion-mediated drug resistance (CAM-DR) of many tumors [23]. Different specific fibronectin domains are bound by α_4_β_1_ and α_5_β_1_ integrins and could each induce opposing effects on cell survival and proliferation [24]. The aim of our study was thus to determine the respective roles of α_4_β_1_ and α_5_β_1_ in GSK3β activation in serum-starved adherent leukemic cells. Our results demonstrate that α_5_β_1_ but not α_4_β_1_ regulates a signaling pathway leading to GSK3β activation and cell survival.

## Materials and Methods

### Antibodies and pharmacological inhibitors

Monoclonal antibodies against GSK3β, flotillin and RACK1 were from BD Transduction Laboratories. Monoclonal antibodies GSK3α/β, actin and integrin subunits (α_5_, P1D6; α_4_, P4G9) were purchased from Upstate or Biosource International (Camarillo, CA, USA), Sigma and Dako (Carpinteria, CA, USA), respectively. Monoclonal antibodies against α_5_ subunit (clone JBS5), Akt and caspases were from Chemicon International, Santa Cruz Biotechnology (Santa Cruz, CA, USA) and Cell Signaling technology (Beverly, MA, USA), respectively. Polyclonal antibodies directed against PP2A-A (catalytic subunit of PP2A) and PP2A tyrosine phosphorylated at position 307 were from Santa Cruz Biotechnology, and those against integrin subunits (α_4_ and α_5_) came from Chemicon International. Polyclonal antibodies directed against PP2A-B' (regulatory subunit of PP2A), cytochrome C, GSK3α/βserine phosphorylated at position 21/9 and Akt threonine phosphorylated at position 308 were from Cell Signaling Technology. Polyclonal antibody against p85 was from Upstate. Horseradish-peroxydase-conjugated secondary antibodies against mouse, rabbit or goat were from Cell Signalling Technology. Okadaic acid, a PP2A inhibitor, and the GSK3β inhibitor SB216763 were from Sigma. For Western blotting after immunoprecipitation, GSK3β (monoclonal from BD Transduction Laboratories) and P(ser9)GSK3β (polyclonal from Abcam) antibodies have been biotynylated in our laboratory.

### Cells and cell culture

The human leukemic cell lines U937, HL-60 and KG1 were purchased from the German Collection of Microorganisms and Cell Cultures (Braunschweig, Germany). U937 and HL-60 cells were grown at 37°C in 5% CO_2_ in RPMI-1640, containing 10% FCS, 50 µg/ml penicillin, and 50 µg/ml streptomycin. KG1 cells were grown in the same conditions in IMDM 20% FCS. Bone marrow leukemic cells from patients with acute myeloid leukemia (AML) were obtained upon informed consent and processed for their conservation as described previously [8]. Leukemic samples were characterized at the Hematology Department of Toulouse University Hospital (France), classified along French American British (FAB) classification (FAB0: undifferentiated AML; FAB1: myeloblastic AML; FAB2: myeloblastic with differentiation AML; FAB4: myelomonocytic AML; FAB5: monocytic AML). The samples contained more than 80% leukemic blasts after processing. After thawing, viable cells from patients were checked by blue trypan labelling, resuspended in IMDM, then washed once and quickly used for in vitro experiments.

### Transfection of siRNA

U937 cells were transfected using the Amaxa nucleofection technology (Amaxa, Koeln, Germany), as indicated in [8]. 6×10^6^ U937 cells in 100 µl solution V were mixed with 200 nM siRNA GSK3β, 100 nM siRNA PP2A-B' or with 100–200 nM non-targeting siRNA (Dharmacon Inc., Lafayette, CO, USA). For siRNA integrin, two sources have been used to target α_4_ and α_5_ subunits: Ambion (30 nM) and Qiagen (50 nM). Cells were immediately nucleofected with an Amaxa Nucleofector apparatus (Amaxa, program V01), then transferred into wells containing 37°C prewarmed culture medium in six-well plates. After transfection, cells were cultured from 24 to 96 h before analysing by Western blotting or FACS. Decrease of GSK3β and α_4_ integrin subunit was maximal at 48 h and maintained at 72 h whereas decrease of PP2A-B' and α_5_ integrin subunit was maximal at 72 h. Therefore, survival tests and Western blot analysis were performed at 72 h post-nucleofection.

### Western blotting

For Western blotting, 0.5–1×10^6^ cells washed in Phosphate Buffer Saline (PBS) were denatured in Laemmli sample buffer. After sonication for 10 seconds and boiling for 10 min, proteins were resolved on polyacrylamide SDS gels (SDS–PAGE) and transferred to nitrocellulose (membrane Hybond-C super, Millipore). The membrane was blocked for 1 h at room temperature in Tris-buffered saline (TBS) containing 5% fat-free milk and then was probed overnight at 4°C with the appropriate monoclonal or polyclonal antibodies in TBS, 0.1% Tween, 3% fat-free milk and 3% Bovine Serum Albumin (BSA, Euromedex). After incubation for 1 h at room temperature with either anti-mouse or anti-rabbit IgG antibody coupled to horseradish peroxidase, or streptavidin-HRP, detection was achieved using a chemiluminescent substrate (SuperSignal, Amersham Pharmacia Biotech).

### Survival and adhesion assays

We have previously set-up a protocol to study the survival pathway in leukemic cells strictly dependent on the integrin engagement without extrinsic growth factors that should not be found in the leukemic niche [8]. Thus, adhesion assays and treatments of cells were performed during 5 h of serum starvation followed by re-addition of serum to discard serum deprivation-linked cytotoxicity, and cell viability was measured at 24 h. Since adhesion under serum-starved conditions was required to trigger GSK3β activation downstream of integrin engagement (preliminary experiments not shown), we have thus measured the differential of GSK3β-linked cell survival between suspension and adhesion upon serum-starved conditions. Half of a 96 well microtiter plate (MaxiSorp Immuno Plate, Nunc, Denmark) was coated overnight at 4°C with 40 µg/ml of human fibronectin (Roche Molecular Biochemicals, Mannheim, Germany) in a final volume of 50 µl in PBS, and subsequently incubated with 1% fatty acid-free BSA in PBS to block non specific adhesion sites, 1 h at room temperature. Leukemic cells were diluted to 0.3×10^6^/ml, left overnight (cell lines) or immediately processed after thawing (AML cells, protocol described below) and then serum-starved for 1 h, incubated or not with okadaic acid (100 nM) or SB216763 (10 µM). Then, cells were allowed to adhere on fibronectin-coated microtiter 96 well plates (0.8×10^5^ cells/well) for 1 h at 37°C or maintained in suspension. Where shown, leukemic cells in suspension or adhesion were treated with 10 ng/ml TNFα for 4 hours after which cells were washed and incubated in serum-containing medium for 24 h at 37°C. Cell viability was then quantified by methyl thiazolyl tetrazolium (MTT) assay (Sigma). In some experiments, leukemic cells were allowed to adhere on a surface coated with α_4_- or α_5_-specific antibodies (clones P4G9, P1D6, JBS5: 1, 0.1 µg/ml and 0.2 µg/ml for optimal adhesion, respectively) as previously described in [8]. Assays were performed in triplicate. For the quantification of cell adhesion, adherent cells were washed in PBS, fixed with a Karnovsky solution and stained with 0.1% crystal violet solution.

For the apoptosis assay, 1×10^6^ U937 cells treated with control, GSK3β or integrin siRNAs were processed as described for the survival assay and at 5 h of incubation were washed with 1x PBS and then incubated 15 min at room temperature with a FITC-labeled Annexin-V/propidium iodide solution (Sigma). These cells were directly analysed in a FACScan (Becton Dickinson) with a sample size of at least 10,000 cells gated on the basis of forward and side scatter. Storing and processing of data was accomplished using FACScan software and allowed the determination of the percentages of living, apoptotic and necrotic cells. Following the protocol described above including adhesion and TNF treatment upon serum starvation, the apoptotic effectors (caspases, cytochrome C) were detected by Western blot at 4 h after re-addition of serum.

In our experiments, we have only used samples from AML patients that did not display cell death after thawing over 10% as checked by blue trypan labeling. After thawing, cells were cultured in IMDM without serum for 2 h and then allowed to adhere on fibronectin. At the end of 4 h adhesion, culture medium was supplemented by SVF to maintain the cells until 24 h. Cell survival of AML blasts from patients was measured at 24 h by MTT labeling. Measurement of apoptosis in AML blasts treated or not by TNFα was realized by FACS using labeling by APO2.7 (APO2.7–PC5 monoclonal antibody from Immunotech, Marseille, France), a mitochondrial membrane protein expressed during the early stages of apoptosis in relation to the release of cytochrome C outside the mitochondria. APO2.7 labeling has been chosen since in our control apoptotic assays performed with daunorubicin we detected interference with the Annexin-V fluorescence (not shown). In our experimental conditions, spontaneous death after thawing was maximally 20% at 24 h (not shown).

### Subcellular fractionation and α_5_ immunoprecipitation

Culture dishes were coated overnight at 4°C with 40 µg/ml of human fibronectin in a final volume of 10 ml in PBS and subsequently blocked with 1% fatty acid-free BSA in PBS, 1 h at room temperature. 30×10^6^ cells were serum starved for 1 h at 37°C, then allowed to adhere to fibronectin-coated dishes for 1 h at 37°C, or maintained in suspension.

For subcellular fractionation, after washing, cells were pelleted by centrifugation (5 min, 100 *g*) and resuspended in a hypotonic buffer [10 mM HEPES (pH 7.2), 10 mM KCl, 1.5 mM MgCl_2_, 0.1 mM EGTA, 20 mM NaF, 100 µM Na_3_VO_4_, 10 µg/ml aprotinin, 10 µg/ml leupeptin and 1 mM PMSF (Phenylmethanesulfonyl fluoride)] for 30 minutes at 4°C, with shaking. Cells were broken up using a Dounce homogenizer (90 strokes), after which the nuclei were pelleted by centrifugation (10 min, 1100 *g*, 4°C). The nuclei-free supernatant was subjected to a second 13500 *g* centrifugation for 45 min at 4°C to separate the membranes from the cytosolic fractions. The membrane pellets were resuspended in lysis buffer [10 nM Tris-HCl (pH 7.5), 150 mM NaCl, 5 mM EDTA, and 1% Triton X-100] and sonicated 1 minute. 50 µg of total cytosolic and membrane pellet proteins were analysed by Western blotting.

For immunoprecipitation of α_5_ integrin, at the end of adhesion, cells were washed in PBS and lysed in a buffer containing 20 mM Tris HCl pH 8, 130 mM NaCl, 1% Triton X-100, 10% glycerol, orthovanadate and protease inhibitors. After sonication and centrifugation at 13500 *g*, supernatant was processed for protein quantification, preclearing and incubation overnight with 20 µL α_5_ polyclonal antibodies. Then, immunoprecipitates were recovered with protein A sepharose, washed and analysed by Western blotting.

### Statistical analysis

Student's t test was used for statistical analysis of *n* independent experiments realized *in vitro*.

## Results

### GSK3β, α_4_β_1_ and α_5_β_1_ integrins are implicated in cell survival of serum-starved adherent leukemic cells

**Figure 1:**
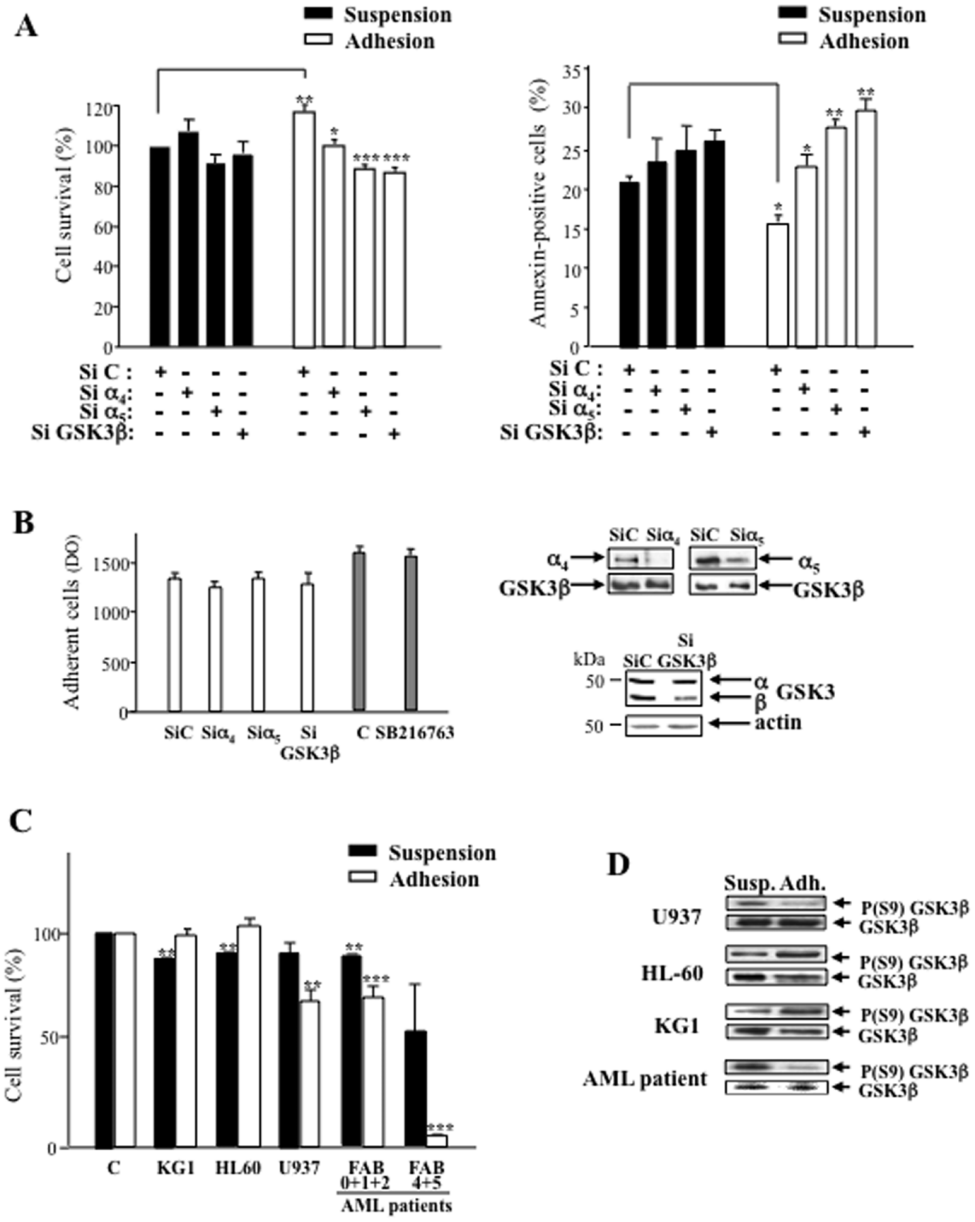
Implication of GSK3β, α_4_β_1_ and α_5_β_1_ integrins in the survival of adherent leukemic cells. **A**- 72 hours after nucleofection of siRNA directed to GSK3β, α_4_ or α_5_ subunits, U937 were serum starved, adhered to fibronectin, and then underwent a survival assay 24 hours later with MTT (left panel, *n* = 6, mean ± S.E.M.) or 5 hours later with annexin-PI labeling (right panel, *n* = 4, mean ± S.E.M.). In MTT assays, data are expressed as percentage of survival compared to the non-targeting siRNA (SiC) in suspension. Statistical analysis on compared adhesion/suspension controls and SiC/SiGSK3 or SiC/SiIntegrin in adhesion: *P<0.05, **P<0.01, ***P<0.001. **B**- The adhesive capacity of siRNA- or SB216763-treated U937 was measured by colorimetry (left panel, *n* = 3, mean ± S.E.M.). SiRNA efficacy against GSK3β, α_4_β_1_ and α_5_β_1_ was checked by Western blot (right panel, representative of three independent experiments). **C**- Survival assay (MTT) was performed as described in Material and Methods with the leukemic cell lines U937, HL-60, KG1 (*n* = 3, mean ± S.E.M.) and the leukemic cells from patients (*n* = 8, mean ± S. E. M.: 1 FAB0, 3 FAB1, 1 FAB2, 1 FAB4, 2 FAB5) after treatment with the GSK3β inhibitor, SB216763 (10 µM). Variations of cell survival in cells treated by SB216763 compared to the untreated cells in suspension or in adhesion are shown: **P<0.01, ***P<0.001. **D**- Variations of the Ser-9 phosphorylation of GSK3β upon adhesion on fibronectin of U937, HL-60, KG1 and AML patient cells (FAB 5) are shown. Representative of three independent experiments.

We have previously demonstrated that α_4_β_1_ and α_5_β_1_ integrins, and the kinase GSK3β, regulate the chemosensitivity of adherent leukemic cells onto fibronectin [8]. Using a siRNA approach, we show that survival of adherent U937 on fibronectin in serum-starved conditions involves both GSK3β and β_1_ integrins (α_4_β_1_ and α_5_β_1_) (Fig. 1A). Decreased expression of GSK3β (70±10%), of α_5_β_1_ (33±10%) and of α_4_β_1_ (47±5%) was assessed by Western blotting (Fig. 1B). None of these siRNA altered the adhesive capacities of leukemic cells (Fig. 1B). Viable cell recovery 24 hours after adhesion assay on fibronectin measured by MTT labeling was increased in adhesion conditions compared to suspension (Fig. 1A, 18±6%, p<0.01). GSK3β and α_5_β_1_ siRNA induced a 30±5% decrease of cell recovery in adhesion (p<0.001), whereas α_4_β_1_ siRNA was less potent (18±5%, p<0.05). No significant changes occurred in suspension upon treatment with the different siRNAs. Of note, identical results were obtained with two sources of siRNA targeting different sequences of α_4_ and α_5_ integrin genes (not shown).

Since MTT measurement could be the result of both cell survival and proliferation, apoptotic cell death was assessed after adhesion assay by annexin labeling (Fig. 1A). Whereas MTT measurement at 24 hours may reflect both apoptotic and necrotic processes, annexin labeling was realized at 5 hours to check specifically for the occurrence of apoptosis. A higher level of apoptosis was detected in U937 electroporated with control siRNA comparatively to untreated cells (20±2% *versus* ≤10%). Adhesion decreased the amount of annexin-positive cells compared to suspension (24±4%, p<0.05). Downregulation of α_5_β_1_ or α_4_β_1_ expression in adherent U937 induced an increase of apoptotic cells compared to control siRNA in adhesion (69±5% p<0.01 and 44±6% p<0.05, respectively). Knockdown of GSK3β expression increased apoptosis of adherent U937 (81±4%, p<0.01). Apoptosis in suspension was not significantly changed after treatment with integrin siRNAs.

Using the pharmacological GSK3β inhibitor SB216763 at a concentration (10 µM) without deleterious quantitative effect on cell adhesion (Fig. 1B), we have further demonstrated that, as well as in U937, the GSK3β-dependent survival pathway occurred in adherent leukemic cells from patients but not in the HL-60 and KG1 leukemic cell lines (Fig. 1C). Accordingly, the activated form of GSK3β (dephosphorylated Ser-9 GSK3β) was increased in adherent U937 and cells from AML patients, but not in adherent HL-60 and KG1 cells (Fig. 1D). Importantly, GSK3β-dependent cell survival was found in adherent AML cells classified along different FAB. However the pro-apoptotic response to SB216763 occurred in 50% AML samples of the cohort (*n* = 16: 3 FAB0, 4 FAB1, 2 FAB2, 3 FAB4 and 4 FAB5) and was more pronounced in AML from myelomonocytic FAB (Fig. 1C). Furthermore, most of the blasts underwent CAM-DR in vitro and SB216763 abolished it, as previously described [8].

Altogether, these results demonstrate that engagement of α_4_β_1_ and α_5_β_1_ integrins to fibronectin supports cell survival of serum-starved adherent leukemic cells, potentially through activation of GSK3β via its dephosphorylation.

### The inhibitory Ser-9 phosphorylation of GSK3β is differentially regulated by α_4_β_1_ and α_5_β_1_ integrins

**Figure 2:**
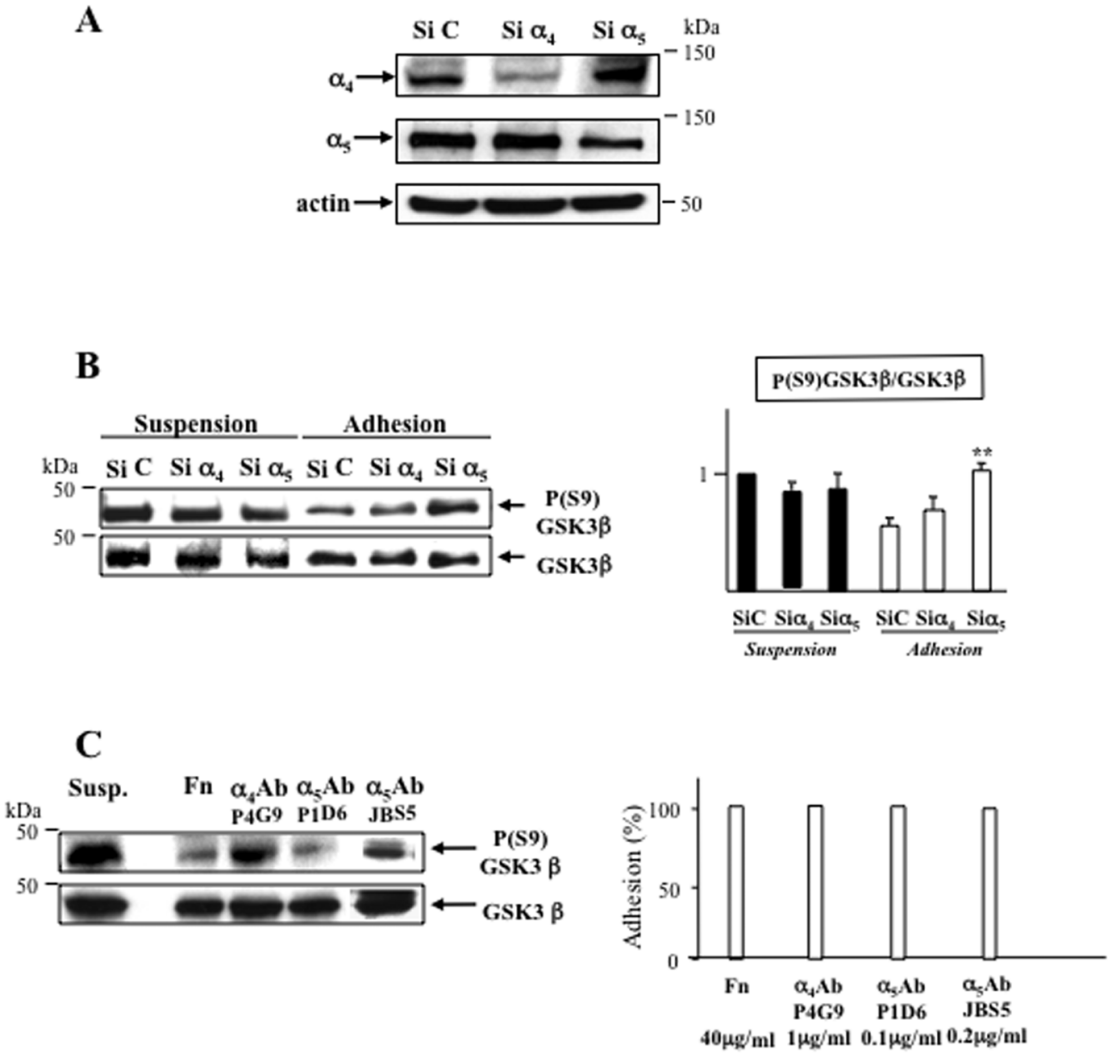
GSK3β is differentially regulated by α_4_β_1_ and α_5_β_1_ integrins in leukemic cells. **A**- Specificity and efficacy of siRNA integrins were checked by Western blot. **B**- Serum-starved U937 were allowed to adhere on fibronectin for 1 h and the Ser-9 phosphorylation state of GSK3β was compared to cells in suspension after downregulation of α_4_ and α_5_ expression by siRNA. SiC = non-targeting siRNA. Right panel shows the mean ± S.E.M. variations of the P(S9)GSK3β/GSK3β ratio analyzed by densitometry from three independent experiments. **C**- The phosphorylation state of GSK3β was studied in suspension or after adhesion of U937 on surfaces coated with fibronectin (Fn, 40 µg/ml), anti-α_4_ (1 µg/ml) or -α_5_ (clone P1D6 0.1 µg/ml; clone JBS5 0.2 µg/ml) antibodies in experimental conditions as for B. Adhesive capacities of U937 in each condition of coating were measured by colorimetry and are shown on right panel. Data are representative of three independent experiments.

To further demonstrate that GSK3β is involved in α_4_β_1_ and α_5_β_1_-mediated prosurvival effect, we used siRNA to knockdown integrin expression (Fig. 2A). We have studied consequences of the decrease in expression of each integrin on GSK3β phosphorylation. In serum-starved conditions, α_4_ and α_5_ siRNA had different effects on the inhibitory Ser-9 phosphorylation of GSK3β (Fig. 2B). Ser-9 phosphorylation of GSK3β was decreased (50%±17, p<0.05) upon adhesion to fibronectin compared to suspension cells. Whereas α_5_ siRNA abolished the Ser-9 dephosphorylation of GSK3β in adhered cells, α_4_ siRNA had no significant effect on Ser-9 phosphorylation. Conversely, in suspension, none of integrin siRNA modified significantly Ser-9 phosphorylation of GSK3β. These results are in favor of GSK3β activation through Ser-9 dephosphorylation after α_5_β_1_ engagement onto fibronectin.

Since we have previously shown that adhesion to surfaces coated with anti-α_4_ (clone P4G9) or -α_5_ (clone P1D6) stimulatory antibodies supported the chemoresistance of U937 cells [8], we studied GSK3β phosphorylation in U937 adhered (with the same efficiency) either to fibronectin or to α_4_ or α_5_ antibodies (Fig. 2C). Adhesion of U937 to coated anti-α_5_ antibodies induced a decrease of Ser-9 phosphorylation of GSK3β compared to suspended cells (60%±10, p<0.01), thus mimicking the observations after adhesion of leukemic cells on fibronectin. However, specific engagement of α_4_β_1_ onto coated anti-α_4_ antibodies did not significantly change the phosphorylation status of GSK3β compared to suspension (10%±15). Interestingly, adhesion of U937 to coated anti-α_5_ antibody clone JBS5 induced a strong adhesion without cell spreading by contrast with fibronectin and clone P1D6 (not shown and [25]) but was not efficient to trigger GSK3β dephosphorylation (Fig. 2C). Of note, adhesion of U937 on non-specific Ig did not induce changes in phosphorylation of GSK3β nor in cell survival (not shown and [8]).

These data unravel a differential control of Ser-9 phosphorylation of GSK3β by α_4_β_1_ and α_5_β_1_ integrins, allowing activation of the enzyme. However, upon adhesion of serum-starved leukemic cells on fibronectin, α_5_β_1_ alone seems to support GSK3β activation.

### Involvement of PP2A in α_5_β_1_-mediated GSK3β activation and cell survival

**Figure 3:**
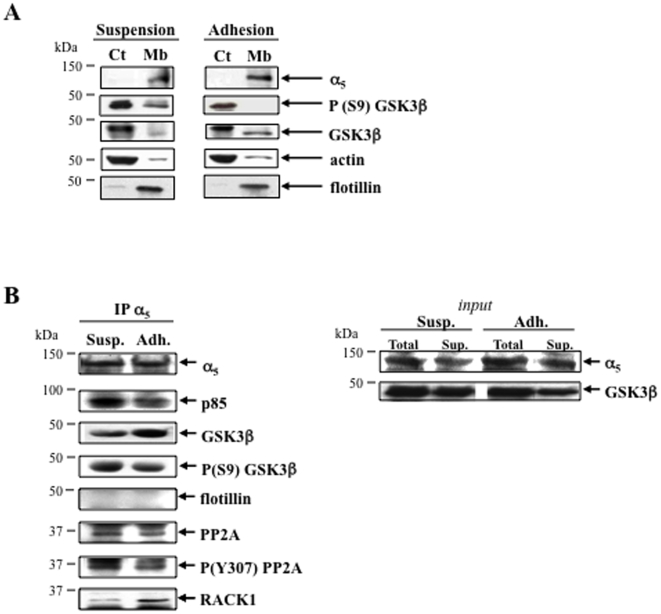
α_5_ integrin, PP2A and GSK3β are co-localized in adherent leukemic cells. **A**- The Ser-9 phosphorylation of GSK3β was studied by Western blot in cytosolic (Ct) and membrane (Mb) compartments of U937 in adhesion or in suspension. **B**- The presence of GSK3β (and its Ser-9 phosphorylated form), PP2A (and its Tyr-307 phosphorylated form), the scaffolding protein RACK1 and the PI 3-kinase regulatory subunit p85 was checked by Western blot in α_5_ immunoprecipitates from U937 in suspension (Susp.) or in adhesion (Adh.). The absence of flotillin in α_5_ immunoprecipitate demonstrates specificity of the interactions between α_5_ integrin, PP2A and GSK3β. Right panel (*input*) indicates the amounts of α_5_ subunit and GSK3β proteins in the total lysates (Total) and in the supernatants (Sup.), before and after immunoprecipitation of α_5_ respectively. Data are representative of three independent experiments.

The phosphatase PP2A is a partner of β_1_ integrins in the control of cell survival [26] and regulates Ser-9 phosphorylation of GSK3β [7]. As shown in Fig. 3A, the inhibitory Ser-9 phosphorylation of GSK3β in the cytosolic/membrane fraction was found to be strongly decreased in adhesion compared to suspension. Moreover, the active form of GSK3β (Ser-9 dephosphorylated GSK3β) was increased in α_5_ integrin immunoprecipitate from adherent U937 comparatively to suspension and was found associated with PP2A (Fig. 3B). PP2A was poorly phosphorylated on its inhibitory site (Tyr-307) in α_5_ immunoprecipitate from adherent U937. Interestingly, adhesion of U937 on fibronectin triggered the association of the scaffolding protein RACK1 with α_5_ whereas it decreased the amount of the PI 3-kinase regulatory subunit p85 associated with the integrin (Fig. 3B). These data show that GSK3β is co-localized with α_5_ integrin in a molecular complex containing phosphatases and kinases potentially implicated in its regulation.

**Figure 4:**
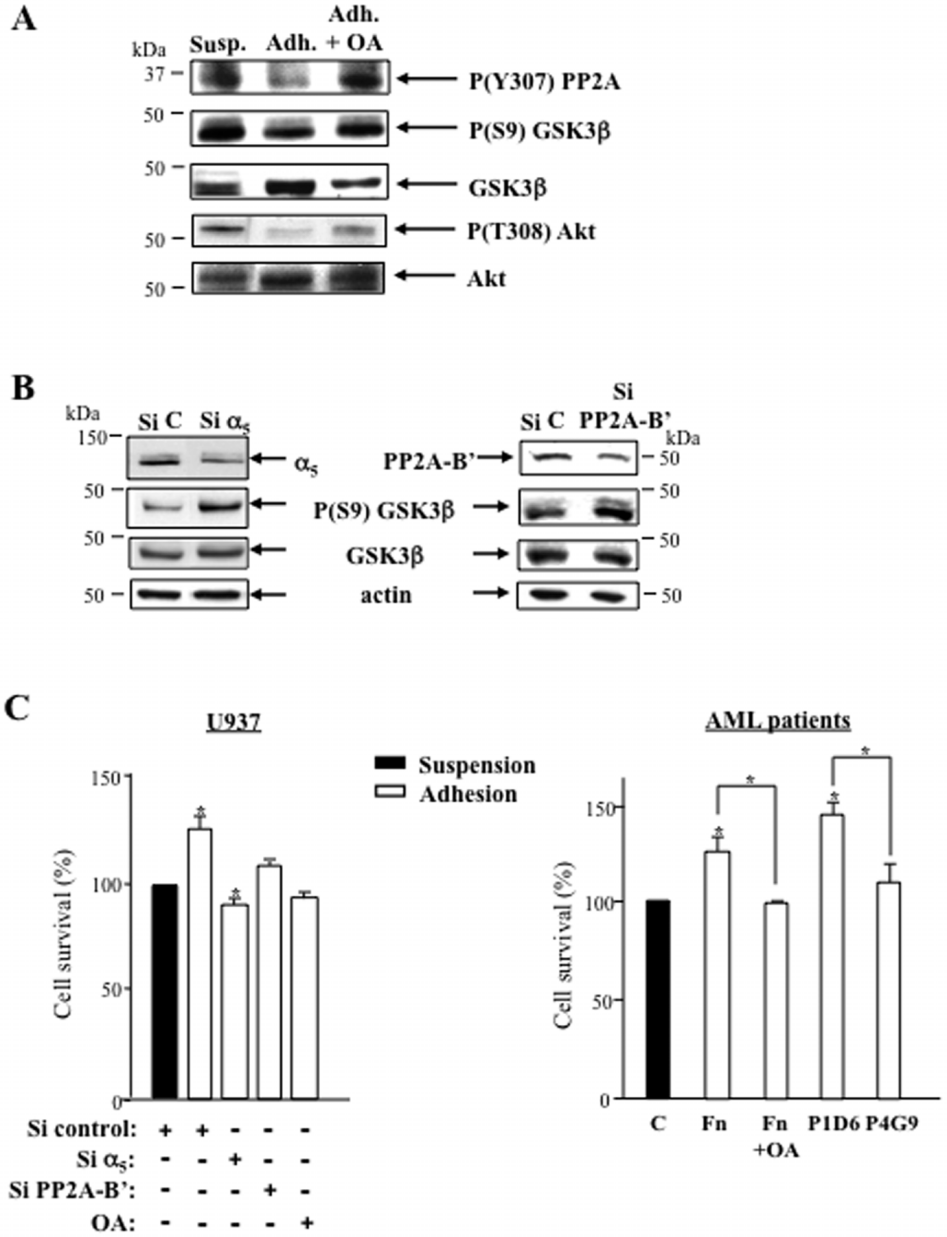
α_5_ integrin and PP2A both regulate GSK3β and survival in leukemic cells. **A**- Variations of inhibited forms of PP2A (P(Y307)) and GSK3β (P(S9)), and activated form of Akt (P(T308)), after adhesion of U937 on fibronectin ± okadaic acid (OA, 100 nM) treatment. **B**- Consequences of α_5_ or PP2A-B' siRNA on Ser-9 phosphorylation of GSK3β were studied. **C**- Adherent U937 transfected with α_5_ siRNA or PP2A-B' siRNA underwent a survival assay (MTT) in serum-starved conditions as described for Fig. 1. Adhesion of cells from AML patients was performed on fibronectin or on α_5_ antibody (P1D6) and α_4_ antibody (P4G9) as described for Fig. 2 to discriminate the respective implication of both integrins in cell survival measured by APO2.7 labeling in the same conditions as for U937. OA (100 nM) was used to inhibit PP2A in U937 and AML blasts. A, B: Data are representative of three independent experiments. In C: U937 *n* = 3 (mean ± S.E.M.), AML patients *n* = 2 (FAB5, mean ± S.D.), comparison to cells in suspension: *P<0.05.

To further demonstrate that PP2A could play a role in GSK3β activation, we used okadaic acid (OA) to inhibit PP2A [26]. The inactive phosphorylated forms of PP2A and GSK3β (phosphoTyr-307 PP2A and phosphoSer-9 GSK3β, respectively) were decreased upon adhesion but restored upon treatment with OA (Fig. 4A) showing that the activation of the two enzymes were correlated. Of note, under treatment by OA, a decrease of GSK3β expression was constantly measured (Fig. 4A) suggesting its Ser-9 phosphorylation-dependent proteasomal degradation as demonstrated previously [27]. The PP2A regulatory subunit B' (also called B56 or PR61) is responsible for the function of PP2A in cytoskeletal stability [28] and Akt regulation [29], potentially involved in the control of cell survival and GSK3β regulation. Indeed we have measured a decrease of the active form of Akt (Threonine 308 phosphorylated Akt) concomitantly with the activation of PP2A and GSK3β in adherent leukemic cells (Fig. 4A). As shown in Fig. 4B, a decrease of α_5_β_1_ and PP2A-B' (40%±5) expression by siRNA both triggered an increase in Ser-9 phosphorylation of GSK3β suggesting their roles in keeping GSK3β in an active state. The PP2A inhibitor OA decreased survival of adherent U937 (35%, p<0.05) to the same extent as α_5_β_1_ knockdown (Fig. 4C). SiRNA directed against the PP2A regulatory subunit B' induced a moderate but significative survival decrease (14%, p<0.05 statistical apparied test) in adhesion (Fig. 4C). In suspension, leukemic cells were not significantly affected under these conditions (not shown). An increase of AML cell survival was measured upon adhesion on fibronectin (27%±6) or α_5_ antibody (clone P1D6, 46%±3) but not α_4_ antibody (clone P4G9, 10%±7) compared to suspension. This improvement of AML cell survival upon adhesion was abolished by OA treatment (Fig. 4C). These results show a role for PP2A in GSK3β activation and cell survival of adherent leukemic cells.

Altogether, these data show that PP2A is activated in serum-starved adherent leukemic cells and cooperate with α_5_β_1_ integrin in leukemic cell survival through the regulation of GSK3β.

### α_5_β_1_ and GSK3β regulate TNFα resistance and both extrinsic and intrinsic apoptotic pathways in leukemic cells

**Figure 5:**
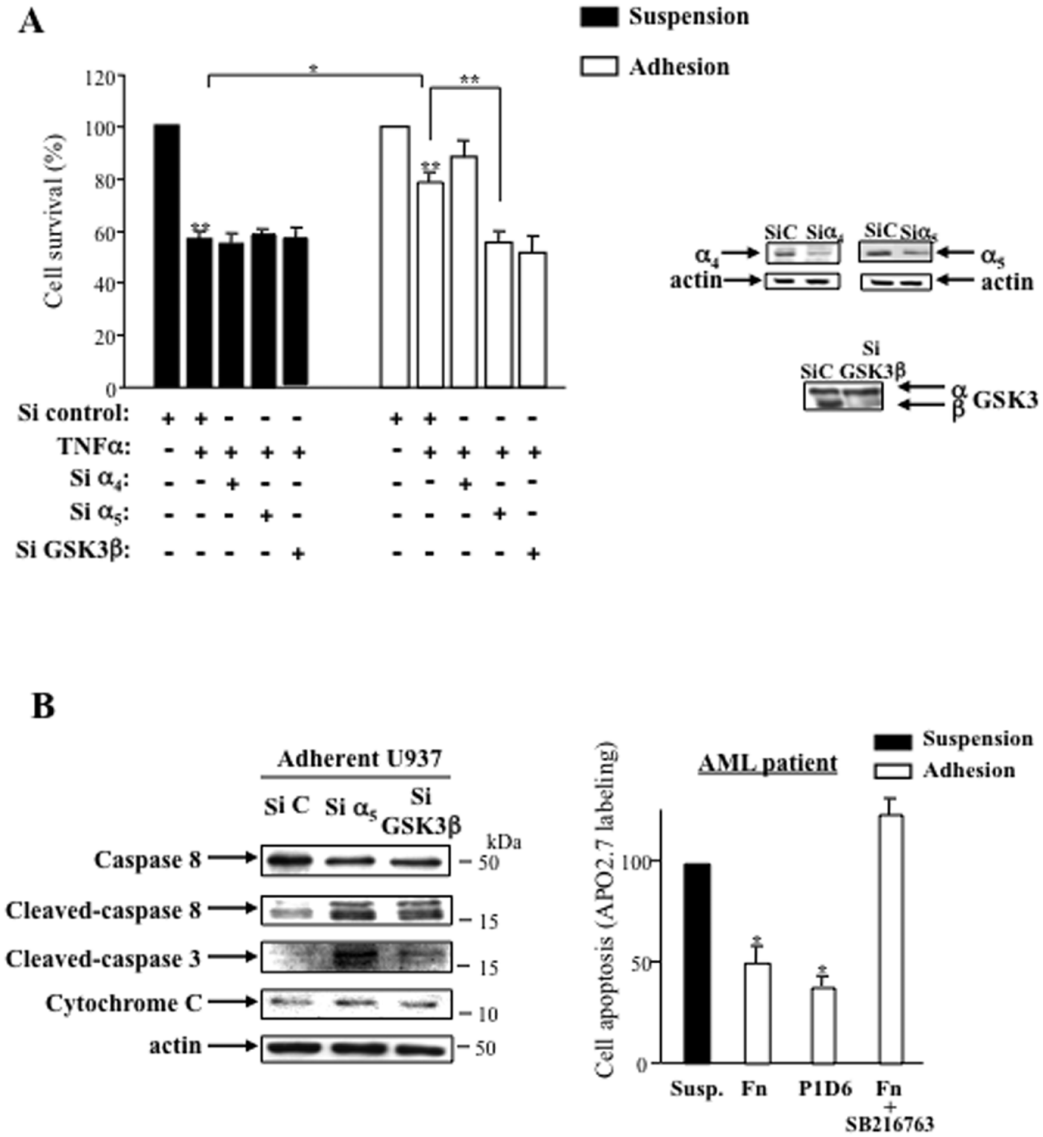
α_5_ integrin and GSK3β regulate the response to TNFα and both extrinsic and intrinsic apoptotic pathways in adherent leukemic cells. **A**- U937 transfected with α_4_, α_5_ or GSK3β siRNA in suspension or adhesion were treated with TNFα (10 ng/ml) for 4 hours and cell survival assays (MTT) were performed as described for Fig. 1. Mean ± S.E.M. *n* = 3, *P<0.05 **P<0.01. Right panel shows the efficacy of siRNA directed to GSK3β, α_4_ and α_5_ integrins. **B**- Extrinsic (caspase-8) and intrinsic (cytochrome C) or both (caspase-3) apoptotic pathways were studied by Western blotting in TNFα-treated adherent U937 treated with siRNA against α_5_ or GSK3β. SiC =  Sicontrol, representative of three independent experiments. On the right side is shown cell apoptosis (APO2.7 labeling) measured in TNFα-treated blasts from AML patients in suspension or in adhesion on fibronectin or on P1D6 α_5_ antibody as described in the legend of Fig. 2 (*n* = 2 FAB5, mean ± S.D., *P<0.05). The treatment with the GSK3β inhibitor SB216763 was peformed as described for Fig. 1.

We have previously shown that both α_5_β_1_ and α_4_β_1_ integrins supported chemoresistance of U937 cells [8]. Since GSK3β activation is α_5_β_1_ integrin-dependent in our conditions and has been shown to play a specific pro-survival role through the response to death receptor activation [21], we checked whether α_5_ and α_4_ integrins could be differentially involved in TNFα response. The incubation of U937 with TNFα in serum-starved suspension conditions induced a 44%±3 (p<0.01) decrease in cell survival (Fig. 5A). Adherent U937 were more resistant to this treatment since a decrease of 24%±4 (p<0.01) of cell survival was measured. α_5_ siRNA as well as GSK3β siRNA, but not α_4_ siRNA, abolished the adhesion-dependent resistance to TNFα(Fig. 5A).

The GSK3β-dependent TNFα resistance and NF-κB activation [8] in adherent leukemic cells suggest that both extrinsic and intrinsic apoptotic pathways could be regulated by α_5_β_1_ integrin engagement. Indeed the treatment of U937 cells with siRNA directed to α_5_ or GSK3β induced a cleavage of caspase 8 and an increase of cytosolic cytochrome C in favor of the activation of both apoptotic pathways in adherent leukemic cells treated by TNFα. As a result of both apoptotic pathways, caspase 3 was cleaved upon this treatment (Fig. 5B). Protection against TNFα confered by α_5_-dependent adhesion and activated GSK3β was confirmed in AML patient (Fig. 5B).

Thus, these data demonstrate that the engagement of α_5_β_1_ integrin and GSK3β activation both support resistance to extrinsic and intrinsic pro-apoptotic pathways of serum-starved adherent leukemic cells.

## Discussion

In this work, we have demonstrated that adhesion to fibronectin triggers a specific survival signaling pathway in U937 leukemic cells upon serum starvation. Importantly, this survival pathway occurs in leukemic blasts from AML patients and supports their chemoresistance [8]. The survival advantage conferred by adhesion to serum-starved leukemic cells requires the activation of GSK3β. A signaling cascade involving the α_5_β_1_ integrin and the phosphatase PP2A is responsible for Ser-9 dephosphorylation and thus activation of GSK3β.

In adherent conditions, co-localization of GSK3β and PP2A with α_5_ integrin in the membrane compartment correlated with Ser-9 dephosphorylation of GSK3β suggesting the activation of the enzyme under these conditions. Indeed, inhibition of α_5_β_1_ and PP2A by siRNA or pharmacological drugs induced an increase of the Ser-9 phosphorylated inhibited form of GSK3β. The β_1_ integrin/PP2A pathway of GSK3β activation may be specific of some integrin heterodimers since it has been demonstrated downstream of α_2_β_1_ [5], α_5_β_1_ but not α_4_β_1_ engagement. Moreover, in our experiments, serum starvation was required to trigger the integrin-dependent GSK3 activation (not shown). The α_5_β_1_/GSK3β pathway supports 30% of survival in adherent leukemic cells. Interestingly, we have previously implicated this survival pathway in cell adhesion-mediated drug resistance, where it was shown to allow a 30% increase in survival [8]. Our data also demonstrate that the α_5_β_1_/GSK3β pathway is involved in TNFα resistance, caspase 8 and cytochrome C regulation, and activation of the transcriptional factor NF-κB [8]. Thus, α_5_β_1_-mediated activation of GSK3β modulates both extrinsic and intrinsic apoptosis signaling pathways [15].

The α_5_β_1_/GSK3β pathway could modulate diverse signaling pathways controlling cell survival. ERK activation has been described to control cell survival that is linked to integrin engagement upon serum starvation [30] or Fas stimulation [26]. Moreover, GSK3β regulates MEKK1 demonstrating its implication in the stress-activated protein kinase pathway [31]. In our experiments, ERK or p38 inhibition did not influence cell survival (not shown). Interestingly, the activity of c-Jun N-terminal kinase (JNK), which is found constitutively activated in most patients with acute myeloid leukemia, has been correlated with a «multidrug» anthracycline resistance [32] and controls fibronectin survival signaling under serum-starvation conditions [33]. Our preliminary data suggest that JNK is activated in adherent U937. It remains to determine whether, in our experimental model, JNK activation is modulated by GSK3β.

Our data show that adhesion of U937 on fibronectin triggers the association of the scaffolding protein RACK1 with α_5_β_1_. Interestingly, RACK1 has been described as a signal integrator between growth factor receptor and β1 integrin [34]. PP2A and the PI 3-kinase regulatory subunit p85 are among the proteins whose recruitment and dissociation are modulated by RACK1. In our experiments, both an increase of activated GSK3β and a decrease of p85 were observed in α_5_ immunoprecipitate from adherent U937. This result suggests that the integrin-dependent activation of GSK3β could result from both increased PP2A and decreased PI 3-kinase/Akt activities [5]. Accordingly we measured a concomitant decrease of the active form of Akt with GSK3β activation in adherent leukemic cells. Thus, survival of U937 in starved conditions may be linked to a quiescence status with decreased proliferative and migration capacities [35]. However, the organization of the cytoskeleton seems to play a key role for the α_5_β_1_-dependent activation of GSK3β since adhesion through the α_5_β_1_ antibody clone JBS5 impaired specifically cell spreading and did not trigger GSK3β activation. Whether RACK1 directly regulates PP2A activity or targets GSK3β and PP2A catalytic/regulatory subunits to specific locations is an open question. Interestingly, it has been shown that RACK1 is a component of the signaling pathway of the p55 TNF receptor [36] and is implicated in the resistance to apoptotic stimuli in hematopoietic cells [37]. Thus, RACK1 could regulate the death-antagonizing complex involving GSK3β at TNF receptor [21].

We have previously shown [8] that engagement of both α_5_β_1_ and α_4_β_1_ supported cell-adhesion mediated drug resistance of U937. However, α_5_β_1_ alone was shown to activate GSK3β and TNFα resistance. Our unpublished data demonstrate that α_4_β_1_ integrin controls U937 survival in adhesion through the tyrosine kinase Pyk2 activation. It suggests that α_5_β_1_/GSK3β and α_4_β_1_/Pyk2-dependent pro-survival pathways could cooperate through the activation of specific pathways of resistance to extrinsic and intrinsic pro-apoptotic pathways. Interestingly, our preliminary data show that the α_4_β_1_/Pyk2 cell survival pathway in adherent U937 involves PI 3-kinase activation and Bcl-xL expression. Since we have shown that the GSK3β-dependent survival pathway occurred in U937 but not in HL-60 and KG1 cell lines, it could be interesting to compare their α_5_ or α_4_-dependent adhesive capacities. Notably, and by contrast with U937, RACK1 increase and PI 3-kinase subunit p85 decrease were not observed in α_5_ immunoprecipitates from adherent HL-60 (not shown). However, we demonstrate that AML cells from patients classified along different FAB trigger mostly the GSK3β-dependent survival pathway upon adhesion onto fibronectin. Except a myelomonocytic phenotype, this survival pathway was not correlated with other clinico-biological parameters in patients.

In conclusion, we propose GSK3β activation as a new adhesion-dependent cell survival pathway that is regulated by the engagement of specific integrin heterodimers. It could control a pro-survival response to death receptors but also intrinsic pro-survival pathways under stress conditions. Importantly, the pro-survival GSK3β-dependent pathway may represent a new therapeutic target in cancer cells whose resistance to therapy is supported by cell adhesion.
